# Effect of Vitamin D_3_ on the Postprandial Lipid Profile in Obese Patients: A Non-Targeted Lipidomics Study

**DOI:** 10.3390/nu11051194

**Published:** 2019-05-27

**Authors:** Salvador Fernández-Arroyo, Anna Hernández-Aguilera, Marijke A. de Vries, Benjamin Burggraaf, Ellen van der Zwan, Nadine Pouw, Jorge Joven, Manuel Castro Cabezas

**Affiliations:** 1 Universitat Rovira i Virgili, Departament de Medicina i Cirurgia, Unitat de Recerca Biomèdica, C/de l’Escorxador S/N, 43003 Tarragona, Spain; jorge.joven@urv.cat; 2 Campus of International Excellence Southern Catalonia, 43003 Tarragona, Spain; 3 Institut d’investigació Sanitària Pere Virgili, C/de l’Escorxador S/N, 43003 Tarragona, Spain; anna.hernandeza@gmail.com; 4 Department of Internal Medicine, Center for Diabetes and Vascular Medicine, Franciscus Gasthuis & Vietland, 3045 PM Rotterdam, The Netherlands; M.deVries@Franciscus.nl (M.A.d.V.); B.Burggraaf@Franciscus.nl (B.B.); M.CastroCabezas@Franciscus.nl (M.C.C.); 5 Department of Clinical Chemistry, Franciscus Gasthuis & Vietland, 3045 PM Rotterdam, The Netherlands; E.vanderZwan@Franciscus.nl (E.v.d.Z.); N.Pouw@Franciscus.nl (N.P.)

**Keywords:** cholecalciferol, lipid absorption, lipidomics, obesity, postprandial inflammation, sphingomyelin

## Abstract

Postprandial lipemia can lead to an accumulation of atherogenic lipoproteins in the circulation associated with systemic low-grade inflammation and an increased risk of cardiovascular disease. Lifestyle and pharmacological treatments are usually prescribed for prevention. Vitamin D_3_ (cholecalciferol), as an anti-atherogenic agent, is being taken into consideration due to its potential beneficial effects in lipid metabolism and its anti-inflammatory potency. To assess the effects of vitamin D_3_ in the postprandial lipid profile in obese, vitamin D-deficient women, a non-targeted lipidomics approach using liquid chromatography coupled to a quadrupole time-of flight mass spectrometer was used to identify and quantitate a wide-range of circulating lipid species, including diglycerides, lysophosphatidylcholines, phosphatidylcholines, phosphatidylethanolamines, sphingomyelins and triglycerides. The most important changes were found in plasmatic sphingomyelin levels, which experience a decrease after vitamin D_3_ intake. Our results suggest a turnover of sphingomyelins, probably due to an increased activity of neutral sphingomyelinases, and, therefore, with implications in the clearance of chylomicrons, LDL and VLDL, decreasing postprandial inflammation and macrophage adherence to endothelia, potentially improving cardiovascular disease risk.

## 1. Introduction

Several forms of evidence suggest that the postprandial period is closely associated to a situation of low-grade systemic inflammation and oxidative stress (1–4). These pathophysiological changes have been linked to diseases like type 2 diabetes mellitus and atherosclerosis. Postprandial lipemia is characterized by the secretion of chylomicrons (CM), VLDL and their remnants. Accumulation of these lipoproteins in the circulation can result in systemic leukocyte activation in addition to impaired endothelial cell function [1,2]. The leukocyte activation starts a signaling cascade where several pro-inflammatory cytokines (IL-1β, IL-6, MCP-1, TNF-α), adhesion molecules (VCAM-1, ICAM-1), integrins (CD11b, CD66b) and the complement system (C3) are involved [2,3,4]. This may lead to migration of leukocytes into the subintimal space of the vessel wall, resulting in foam cell formation and development of the atherosclerotic plaque.

Numerous lifestyle and pharmacological interventions have been described to reduce (postprandial) inflammation and cardiovascular disease (CVD) risk [5]. The intake of dietary polyphenols with antioxidant and anti-inflammatory properties [6,7,8] or a monounsaturated or polyunsaturated fatty acid rich-diet [9,10] seems to diminish postprandial inflammation, to increase insulin sensitivity and to decrease the CVD risk. Likewise, the use of lipid-lowering drugs (such as statins, fenofibrate, rosiglitazone or metformin) have similar effects [11,12,13,14].

Vitamin D_3_ (cholecalciferol), belonging to the family of secosteroids, has received special attention in the last years due to its potential pleiotropic effects on lipid metabolism [15], the modulation of cell proliferation [16], cytokine production [17], immune system function [18,19,20], arterial stiffness [21] and inflammation [22]. In particular, the effects of vitamin D_3_ on mitochondrial oxidation and phospholipid metabolism have received much attention [23,24]. Since phospholipids are major components of the lipoprotein membranes, we were interested in the changes occurring in the human plasma after vitamin D_3_ ingestion. So far, no data on this subject have been published.

The present study describes a double-blind randomized study showing the effects of different doses of vitamin D_3_ on postprandial lipid profile in obese, pre-menopausal, vitamin D-deficient women by means of a non-targeted lipidomics approach, including diglycerides (DG), lysophosphatidylcholines (LPC), phosphatidylcholines (PC), phosphatidylethanolamines (PE), sphingomyelins (SM) and triglycerides (TG).

## 2. Material and Methods

### 2.1. Reagents

Grade MS methanol (MeOH) and 2-propanol, ammonium formate, formic acid, acetic acid, methyl *tert*-buthyl ether (MTBE) and standards for calibration curves (1,3-dilinoleoyl-*rac*-glycerol [DG 36:4], 1-palmitoyl-2-oleoyl-3-linoleoyl-*rac*-glycerol [TG 52:3], 1-oleoyl-2-hydroxy-*sn*-glycero-3-phosphocholine [LPC 18:1], 1,2-dioleoyl-*sn*-glycero-3-phosphocholine [PC 36:2], 1,2-dipalmitoyl-*sn*-glycero-phosphoethanolamine [PE 32:0] and N-palmitoyl-d-erythro-sphingosylphosphorylcholine [SM 34:1]) were purchased from Sigma Aldrich (Saint Louis, MO, USA). Water (milliQ grade) was obtained from a Milli-Q integral water purification system (Millipore Corp., Burlington, MA, USA). For internal standards, SPLASH Lipidomix was purchased from Avanti Polar lipids (Alabaster, AL, USA).

### 2.2. Subjects and Study Design

A 1:1 randomized, double-blind trial was designed to compare the lipid profile on postprandial inflammation in obese, pre-menopausal, vitamin D-deficient women (*n* = 24) after a single high (300,000 IU) (*n* = 12) or low (75,000 IU) (*n* = 12) dose of cholecalciferol supplementation. The study was carried out following the rules of the Declaration of Helsinki of 1975 (revised in 2013) and was approved by the Institutional Review Board of the Franciscus Gasthuis, Rotterdam, the Netherlands, and the regional independent medical research ethics committee (TWOR), Rotterdam, the Netherlands. All participants provided written informed consent. The study was registered at clinicaltrials.gov under the number NCT01967459. Inclusion and exclusion criteria and clinical characteristics have been published previously [25]. Blood samples were drawn from the participants after a 10-h overnight fast (baseline). Then, 50 g of fat (commercial fresh cream, Albert Heijn, Zaandam, the Netherlands) per m^2^ body surface was ingested (the composition of fresh cream consisted of 35% of fat, of which 23% were saturated, 10% monounsaturated and 0.9% polyunsaturated). Blood samples were collected at 2, 4, 6 and 8 h after ingestion. During the fat load test, patients were only allowed to drink water. At the end of the first oral fast loading test, participants received a single dose of cholecalciferol (double-blinded for high and low dose). The oral fat loading test was repeated after 7 days following the same protocol.

### 2.3. Samples,External Calibration Curve and Quality Control Preparation

Lipid extraction was performed with a modification of the Bligh and Dyer protocol [26]. Briefly, 750 μL of a solution consisting of MTBE:MeOH (1:2 *v*/*v*) with 0.5% acetic acid and containing 1:100 (*v*/*v*) internal standard mixture (SPLASH Lipidomix) were added to 20 μL of plasma and vortexed for 10 min. Afterwards, 250 μL of MTBE and 350 μL of water were added, with a vortex step of 1 min between both additions. After centrifugation at 15000 rpm during 10 min at 4 °C, 350 μL of organic phase were collected, dried in a Savant SPD2010 SpeedVac rotatory vacuum system (Thermo Fischer, Waltham, MA, USA), reconstituted in 100 μL of MeOH:MTBE (9:1 *v*/*v*) and placed into glass vials for LC-MS analysis.

For calibration curves, stock standards were dissolved in MeOH. Then, 10 seriated concentrations (range from 0.2 to 100 μM) containing 1:100 (*v*/*v*) internal standard mixture were prepared dissolving stocks in MeOH:MTBE (9:1 *v*/*v*) and placed into glass vials for LC-MS analysis. Calibration curves were plotted using the relative response (ordinate axis) and relative concentration (abscisse axis) to the internal standard. Slope and linearity for each standard are detailed in Supplementary Table S1.

For quality control, a pool of different samples included in the study was made, lipids were extracted and injected twice a day during the analysis to ensure the reproducibility of the experiment. Overlaid chromatograms of quality controls are available in Supplementary Figure S1.

### 2.4. UHPLC-ESI-QTOF-MS Conditions

Samples (2 μL) were injected directly into a 1290 Infinity ultra-high-pressure liquid chromatograph (UHPLC) coupled with a dual Agilent jet stream electrospray ionization (ESI) source to a 6550 quadrupole-time-of-flight mass spectrometer (QTOF) (Agilent Technologies, Santa Clara, USA). The UHPLC system was equipped with a binary pump (G4220A), an autosampler (G4226A) termostatized at 4 °C, and a Kinetex EVO C18 column 2.6 µm, 2.1 mm × 100 mm (Phenomenex, Torrance, CA, USA). The mobile phase consisted of A: water, B: MeOH and C: 2-propanol containing 10 mM ammonium formate +0.1% formic acid, at a flow rate of 0.6 mL/min. The gradient used was as follows: 0 min, 10% B, 40% C; 0.5 min, 10% B, 50% C; 1.5 min, 9.5% B, 52.5% C; 1.6 min, 7.5% B, 63.5% C; 5 min, 7% B, 66.5% C; 5.1 min, 4% B, 82.5% C; 7.5 min, 3.5% B, 85% C; 9 min, 3.5% B, 85% C; 9.5 min, 0% B, 100% C; 11.5 min, 0% B, 100% C; 11.6 min, 10% B, 40% C. A post run of 2 min in initial conditions was used for column conditioning. For the ESI source, the optimized parameters were as follows: gas temperature 225 °C, gas flow 11 L/min, nebulizer 35 psi, sheath gas temperature 300 °C, and sheath gas flow 12 L/min. For the QTOF-MS, running in positive mode, the capillary, nozzle and fragmentor voltages were set at 3500 V, 500 V and 380 V, respectively.

### 2.5. Data Analysis and Statistics

Prior to the injection of the samples into the LC-MS system, a pool of samples was injected and the raw chromatogram was deconvoluted using “Find by Molecular Feature” algorithm from MassHunter Qualitative Analysis B.07.00 software (Agilent Technologies, Santa Clara, CA, USA). Lipid characterization was performed by matching their accurate mass and isotopic distribution to Metlin-PCDL database (Agilent Technologies, Santa Clara, CA, USA) allowing a mass error of 10 ppm and a score higher than 80 for isotopic distribution. To ensure the tentative characterization, chromatographic behavior of pure standards and corroboration with Lipid Maps database (www.lipidmaps.org) was carried out. Afterwards, compound match entities were selected to perform a targeted MS/MS acquisition on LC-QTOF-MS instrument to corroborate the identification. It is important to note that previous identification of compounds before statistical analysis allows us to use the most appropriate internal standards based on the lipid family to correct the instrumental deviations and increase the statistical power of the study. Suplementary Table S2 provides *m*/*z* and retention time values of each lipid species characterized.

Selected lipid species were indirectly quantitated across all the samples using MassHunter Quantitative Analysis B.07.00 (Agilent Technologies, Santa Clara, CA, USA) attending to the calibration curve of its corresponding standard.

Outliers were detected using the interquartile range method. Missing values and outliers were replaced by the median of the group and data was normalized using the log_2_. Paired or unpaired *t*-tests with Bonferroni correction were used to compare groups using SPSS 25 (IBM Corp., Armonk, NY, USA) with a significance threshold of 0.05. Partial least square discriminant analysis (PLS-DA) and heatmaps were performed in Metaboanalyst 4.0 (www.metaboanalyst.ca) [27] using the already normalized database (thus, without further steps of data processing in Metaboanalyst).

## 3. Results

### 3.1. Characterization, Quantitation and Absorption Dynamics of Lipid Species

**Figure 1:**
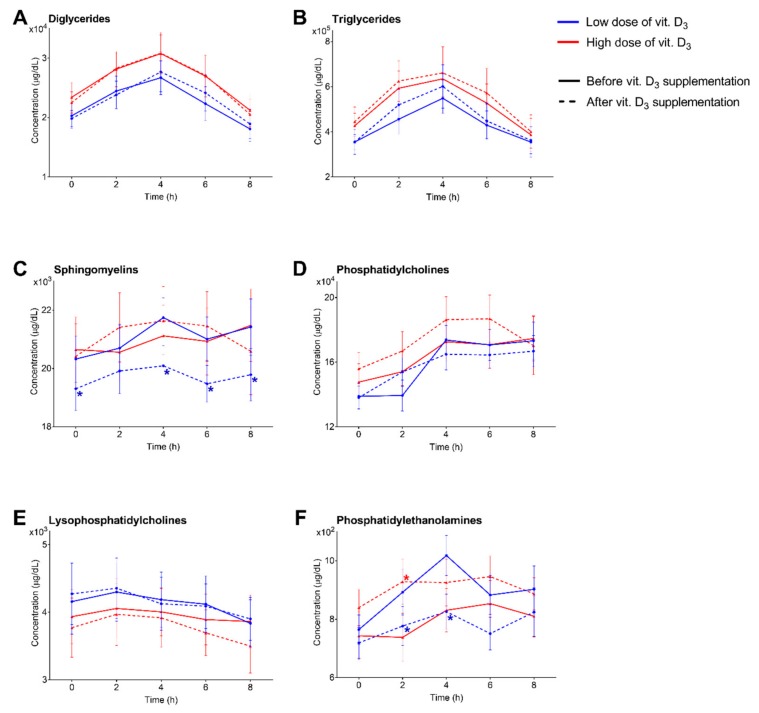
Concentrations (in μg/dL) of lipid species grouped by families (expressed as mean ± SEM) during the postprandial fat intake before and after vitamin D3 supplementation. (**A**) diglycerides; (**B**) triglycerides; (**C**) sphingomyelins; (**D**) phosphatidylcholines; (**E**) lysophosphatidylcholines; (**F**) phosphatidylethanolamines. HDL, high-density lipoprotein; LDL, low-density lipoprotein; DG, diglycerides; LPC, lysophosphatidylcholines; PC, phosphatidylcholines; PE, phosphatidylethanolamines; SM, sphingomyelins; TG triglycerides. *p*-value < 0.05 between day 1 and day 7 in low dose (blue asterisc) and high dose (red asterisc) groups.

**Figure 2:**
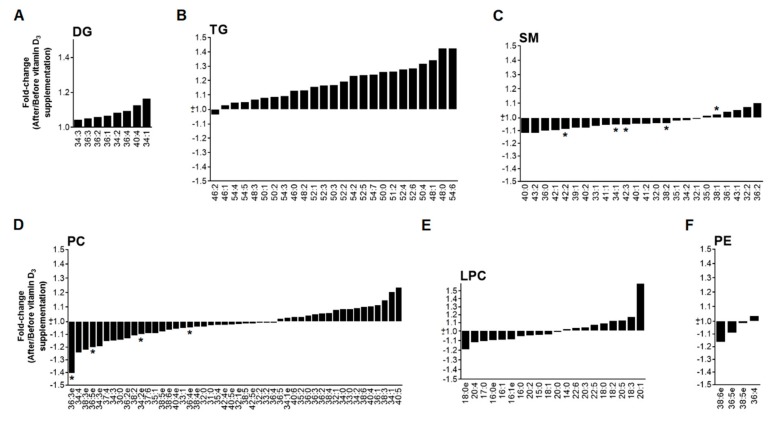
Fold-change (after/before) at time 0 h of lipid species (expressed as mean) in the low dose of vitamin D_3_ group. (**A**) diglycerides; (**B**) triglycerides; (**C**) sphingomyelins; (**D**) phosphatidylcholines; (**E**) lysophosphatidylcholines; (**F**) phosphatidylethanolamines. * *p*-value < 0.05.

**Figure 3:**
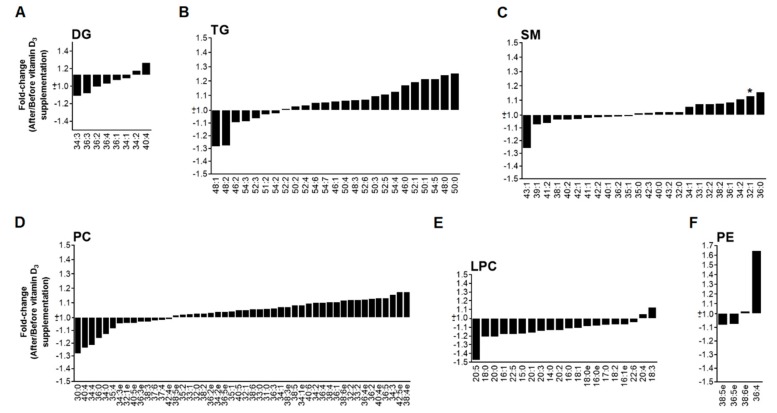
Fold-change (after/before) at time 0 h of lipid species (expressed as mean) in the high dose of vitamin D_3_ group. (**A**) diglycerides; (**B**) triglycerides; (**C**) sphingomyelins; (**D**) phosphatidylcholines; (**E**) lysophosphatidylcholines; (**F**) phosphatidylethanolamines. * *p*-value < 0.05.

A total of 128 lipids belonging to 6 different families were characterized, including 8 DG, 20 LPC, 47 PC, 4 PE, 24 SM and 25 TG. Concentrations of all lipid species can be found in the Supplementary Table S3. The most important baseline variables, as well as lipid concentrations (grouped by family), at the beginning of the study for the low and high vitamin D_3_ supplementation groups are listed in Table 1. Interestingly, some specific lipids (DG 36:1, DG 36:2, TG 48:0, TG 50:0 and TG 52:1) were statistically different at the beginning of the study (Supplementary Table S3). Lipidomics experiment at day 1 (before vitamin D_3_ treatment) revealed a maximum absorption of DG, PC, SM and TG at 4 h after the fat intake, 2 h for LPC and between 4 and 6 h for PE. A similar pattern of absorption dynamics was found at 7 days after vitamin D_3_ supplementation (Figure 1), with some differences depending to the dose administrated (Figure 2 and Figure 3), as detailed below.

### 3.2. Low Dose of Vitamin D_3_ Decreases Total SM Levels and Specific PC and PE Species

**Figure 4:**
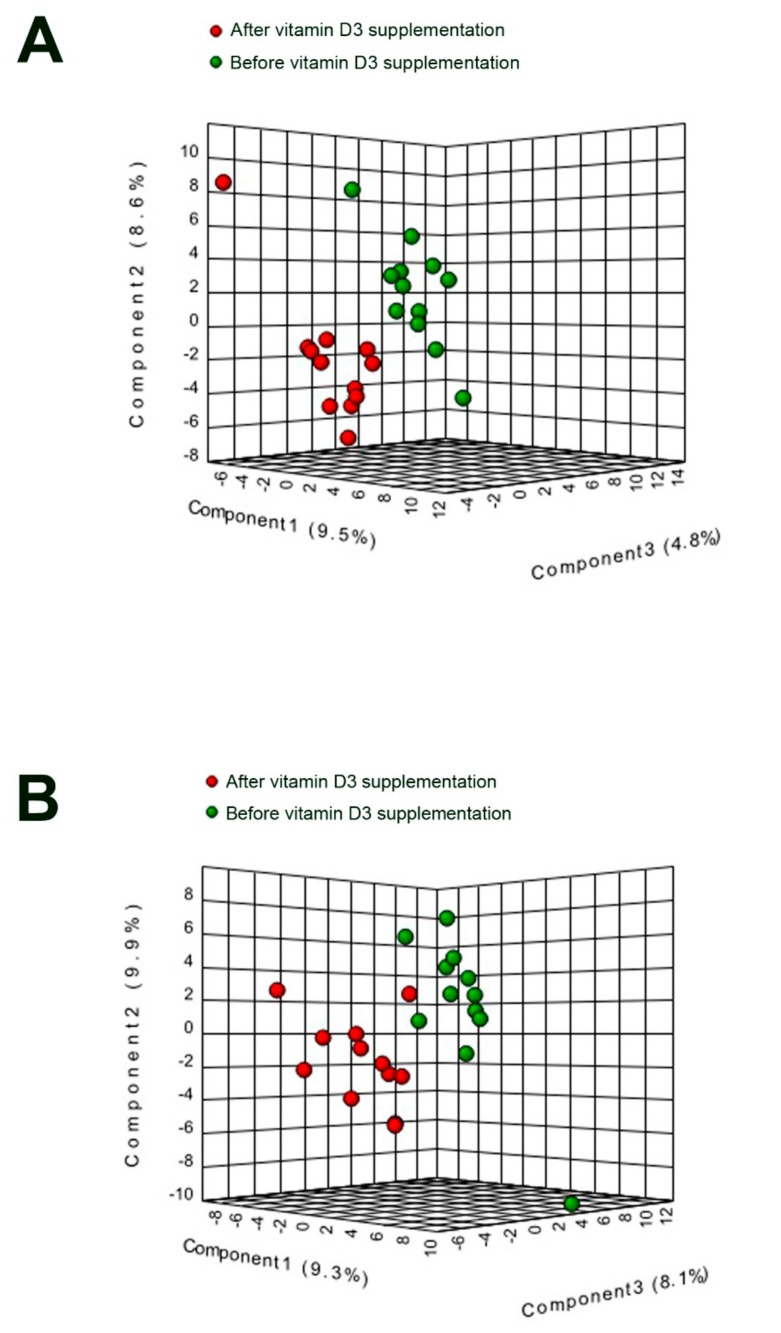
Partial least square discriminant analysis of patients before (green) and after (red) vitamin D_3_ intake in the low (**A**) and high (**B**) dose groups.

Comparing the lipid values before and after vitamin D_3_ supplementation, we observed statistically significant changes in the circulating lipids (Supplementary Table S3). Even if the total concentration of PC were not significantly reduced, we found a decrease in several lipids belonging to this family, mainly in the alkyl ether species (Figure 2). In the same way, absorption of PE was also decreased after vitamin D_3_ supplementation at 2 and 4 h after a fat loading test (Figure 1F and Figure 2F). Of note, the most important differences were found in the SM family, including a decrease at day 7 in almost the entirety of the SM species quantitated (Figure 1C and Figure 2C). No significant changes were found in levels of DG, TG or LPC before and after vitamin D_3_ treatment (Figure 1A,B,E and Figure 2A,B,E). Differences between day 1 and day 7 after vitamin D_3_ supplementation at time 0 h allow us to separate both groups in a PLS-DA analysis (Figure 4A). However, no characteristic patterns can be deduced in the heatmap (Supplementary Figure S2A).

### 3.3. High Dose of Vitamin D_3_ Increases Specific PC and PE Species

Similar to the low dose group, the total PC concentration remained unchanged before and after vitamin D_3_ supplementation (Figure 1D and Figure 3D), but, in contrast, several specific PC species were increased (Supplementary Table S3) after the oral fat intake, especially those with high-degree of unstaturations. PE levels were only significantly increased at 2 h after the fat loading test (Figure 1F). No significant changes were found in total levels of DG, TG, SM or LPC (Figure 1A–C,E and Figure 3A–C,E). In the same way than patients with a low dose of vitamin D_3_, PLS-DA separates both groups (Figure 4B), but any characteristic pattern can be observed in the heatmap (Supplementary Figure S2B).

## 4. Discussion

CVD, obesity and diabetes (closely related metabolic diseases) are becoming one of the most important epidemics of the 21th century, with alarming growth occurring in the developed societies. Dyslipidemia, insulin resistance or inflammation, among other factors, due to a sedentary lifestyle and unhealthy diet promote a mitochondrial impairment and, thus, an imbalance between energy intake and expenditure leading to metabolic disturbances and disease [28]. In the last decade, several studies focused on the actions of vitamin D, either directly or via its receptor. The impact of cholecalciferol in lipid metabolism is well known, but the underpinned mechanisms still remain unclear [15,29,30,31].

In our study, we analyzed different species of DG, LPC, PC, PE, SM and TG families. To the best of our knowledge, this is the first time that an interventional study explored the postprandial fatty acid profile while including this wide range of lipids in relation to vitamin D. Only a significant decrease of total SM and specific PC and PE levels were found after a low dose of vitamin D_3_ supplementation. SM, PC and PE are related through the same biosynthetic pathway, being phosphorylcholine from PC or phosphorylethanolamine from PE precursors of SM synthesis [32]. However, our results are not sufficient to support the idea that a decrease in SM levels is due to a lack of precursors, because only 4 PE species were detected and quantitated (we cannot ensure that PE levels globally decrease) and only a reduction in specific PC species, but not in the total PC content, was found.

This SM turnover by vitamin D_3_ was already observed in vitro using HL-60 cells, for the first time, by Okazaki et al. in 1989 [33]. It is important to note that intracellular changes are not necessarily reflected in plasma. Moreover, an inter-organ metabolic crosstalk should be considered in these patients [34,35]. However, according to the authors, and analogous to our results, we can speculate that cholecalciferol decreases SM levels while PC levels remain similar, with an increase in ceramide and phosphorylcholine units, suggesting that the hydrolysis of SM induced by vitamin D_3_ could be through activation of neutral SMases. Similar results were found in the human keratinocytes HaCaT cell line [36,37] and in glioblastoma cells [38].

SM in plasma is mainly associated to apo B-containing lipoprotein particles, including CM and VLDL [39]. SM are also important components of cell membranes and regulate cell growth, differentiation and apoptosis [40], being also involved in the adherence during macrophage differentiation and adherence [41], cholesterol distribution and homeostasis [42] and in the development of Niemann-Pick disease [43] and increased CVD risk [44].

In contrast to our expectations, a high dose of vitamin D_3_ reverted the effects observed in the low dose group. These data suggest a possible loss of effectiveness when exceeding the recommended dose. The increasing use of vitamin D, inappropriately prescribed, or due to the abuse of new nutraceuticals is a subject of major debate to ensure efficacy and safety in dose and administration guidelines [45,46,47,48].

In conclusion, we describe, for the first time in humans, the effects of vitamin D_3_ in the profile and absorption dynamics of a wide range of lipid species after a fat intake using a non-targeted lipidomics approach. Our results show that a low dose (75,000 IU) of cholecalciferol induces an SM turnover, probably due to the activation of neutral SMases, which can be of interest due to its implications in the clearance of CM, LDL and VLDL, mitigating the postprandial inflammation, the macrophage adherence to endothelia and ameliorating CVD risk. In contrast, high doses of vitamin D_3_ can have undesirable effects in the lipid profile.

## Figures and Tables

**Table 1:** Baseline characteristics of the participants. Data are expressed as mean ± SEM.

	Low Dose Vit. D_3_ (*n* = 12)	High Dose Vit D_3_ (*n* = 12)
Age (years) ^a^	29 ± 3	27 ± 2
BMI (Kg/m^2^) ^a^	31.2 ± 1.3	33.0 ± 1.0
Glucose (mmol/L) ^a^	5.2 ± 0.1	5.2 ± 0.1
Total cholesterol (mmol/L) ^a^	5.1 ± 0.2	5.3 ± 0.3
HDL-cholesterol (mmol/L) ^a^	1.5 ± 0.1	1.4 ± 0.1
LDL-cholesterol (mmol/L) ^a^	3.1 ± 0.2	3.4 ± 0.3
Leukocyte count (10^9^/L) ^a,^*	6.6 ± 0.3	8.1 ± 0.6
Monocyte count (10^9^/L) ^a^	0.5 ± 0.06	0.5 ± 0.03
Neutrophil count (10^9^/L) ^a,^*	3.4 ± 0.3	4.4 ± 0.4
25-OH vitamin D (nmol/L) ^a^	27.3 ± 4.5	26.8 ± 3.6
DG (μg/dL)	20,312 ± 2095	23,425 ± 2467
LPC (μg/dL)	4158 ± 482	3935 ± 403
PC (μg/dL)	139,244 ± 7972	147,992 ± 11,465
PE (μg/dL)	765 ± 50	744 ± 82
SM (μg/dL)	20,325 ± 793	20,647 ± 1115
TG (μg/dL)	355,163 ± 54,657	427,374 ± 55,708

HDL, high-density lipoprotein; LDL, low-density lipoprotein; DG, diglycerides; LPC, lysophosphatidylcholines; PC, phosphatidylcholines; PE, phosphatidylethanolamines; SM, sphingomyelins; TG triglycerides. ^a^ Data extracted from de Vries et al. [17]. * *p*-value < 0.05.

## Supplementary Materials

The following are available online at https://www.mdpi.com/2072-6643/11/5/1194/s1. Experimental *m*/*z* and retention time of each lipid species quantitated are available on Table S1; slope and linearity of standards used for the external calibration curves are available on Table S2; concentration of each lipid species analyzed is available on Table S3; overlaid chromatograms of quality controls are available on Figure S1; heatmap of patients before and after vitamin D_3_ intake on Figure S2.

## References

[1] De Vries M.A., Klop B., Alipour A., van de Geijn G.J., Prinzen L., Liem A.H., Valdivielso P., Rioja Villodres J., Ramirez-Bollero J., Castro Cabezas M. (2015). In vivo evidence for chylomicrons as mediators of postprandial inflammation. Atherosclerosis.

[2] De Vries M.A., Klop B., Janssen H.W., Njo T.L., Westerman E.M., Castro Cabezas M. (2014). Postprandial inflammation: Targeting glucose and lipids. Adv. Exp. Med. Biol..

[3] Alipour A., Elte J.W.F., van Zaanen H.C.T., Rietveld A.P., Castro Cabezas M. (2007). Postprandial inflammation and endothelial dysfunction. Biochem. Soc. Trans..

[4] Teeman C.S., Kurti S.P., Cull B.J., Emerson S.R., Haub M.D., Rosenkranz S.K. (2016). Postprandial lipemic and inflammatory responses to high-fat meals: A review of the roles of acute and chronic exercise. Nutr. Metab..

[5] Klop B., Proctor S.D., Mamo J.C., Botham K.M., Castro Cabezas M. (2012). Understanding postprandial inflammation and its relationship to lifestyle behaviour and metabolic diseases. Int. J. Vasc. Med..

[6] Santhakumar A.B., Battino M., Alvarez-Suarez J.M. (2018). Dietary polyphenols: Structures, bioavailability and protective effects against atherosclerosis. Food Chem. Toxicol..

[7] Cheng Y.C., Sheen J.M., Hu W.L., Hung Y.C. (2017). Polyphenols and Oxidative Stress in Atherosclerosis-Related Ischemic Heart Disease and Stroke. Oxid. Med. Cell Longev..

[8] Yang C.S., Wang H., Sheridan Z.P. (2018). Studies on prevention of obesity, metabolic syndrome, diabetes, cardiovascular diseases and cancer by tea. J. Food Drug. Anal..

[9] Robinson L.E., Buchholz A.C., Mazurak V.C. (2007). Inflammation, obesity, and fatty acid metabolism: Influence of n-3 polyunsaturated fatty acids on factors contributing to metabolic syndrome. Appl. Physiol. Nutr. Metab..

[10] Fritsche K.L. (2015). The science of fatty acids and inflammation. Adv. Nutr..

[11] Verseyden C., Meijssen S., van Dijk H., Jansen H., Castro Cabezas M. (2003). Effects of atorvastatin on fasting and postprandial complement component 3 response in familial combined hyperlipidemia. J. Lipid Res..

[12] Coll B., van Wijk J.P., Parra S., Castro Cabezas M., Hoepelman I.M., Alonso-Villaverde C., de Koning E.J., Camps J., Ferre N., Rabelink T.J. (2006). Effects of rosiglitazone and metformin on postprandial paraoxonase-1 and monocyte chemoattractant protein-1 in human immunodeficiency virus-infected patients with lipodystrophy. Eur. J. Pharmacol..

[13] Rosenson R.S., Huskin A.L., Wolff D.A., Helenowski I.B., Rademaker A.W. (2008). Fenofibrate reduces fasting and postprandial inflammatory responses among hypertriglyceridemia patients with the metabolic syndrome. Atherosclerosis.

[14] Halkes C.J.M., van Dijk H., de Jaegere P.P.T., Plokker H.W.M., van der Helm Y., Erkelens D.W., Castro Cabezas M. (2001). Postprandial increase of complement component 3 in normolipidemic patients with coronary artery disease. Effects of expanded-dose simvastatin. Arterioscler. Thromb. Vasc. Biol..

[15] Silvagno F., Pescarmona G. (2017). Spotlight on vitamin D receptor, lipid metabolism and mitochondria: Some preliminary emerging issues. Mol. Cell. Endocrinol..

[16] Girgis C.M., Clifton-Bligh R.J., Mokbel N., Cheng K., Gunton J.E. (2014). Vitamin D signaling regulates proliferation, differentiation, and myotube size in C2C12 skeletal muscle cells. Endocrinology.

[17] Zhang Y., Leung D.Y., Richers B.N., Liu Y., Remigio L.K., Riches D.W., Goleva E. (2012). Vitamin D inhibits monocyte/macrophage proinflammatory cytokine production by targeting MAPK phosphatase-1. J. Immunol..

[18] Cantorna M.T., Snyder L., Lin Y.D., Yang L. (2015). Vitamin D and 1,25(OH)2D regulation of T cells. Nutrients.

[19] Prietl B., Treiber G., Pieber T.R., Amrein K. (2013). Vitamin D and immune function. Nutrients.

[20] El-Fakhri N., McDevitt H., Shaikh M.G., Halsey C., Ahmed S.F. (2014). Vitamin D and its effects on glucose homeostasis, cardiovascular function and immune function. Horm. Res. Paediatr..

[21] Klop B., van de Geijn G.J., Birnie E., Njo T.L., Janssen H.W., Jansen H.G., Jukema J.W., Elte J.W., Castro Cabezas M. (2014). Vitamin D3 mediated effects on postprandial leukocyte activation and arterial stiffness in men and women. Eur. J. Clin. Nutr..

[22] Querfeld U. (2013). Vitamin D and inflammation. Pediatr. Nephrol..

[23] Boyan B.D., Sylvia V.L., Dean D.D., Pedrozo H., Del Toro F., Nemere I., Posner G.H., Schwartz Z. (1999). 1,25-(OH)_2_D_3_ modulates growth plate chondrocytes via membrane receptor-mediated protein kinase C by a mechanism that involves changes in phospholipid metabolism and the action of arachidonic acid and PGE2. Steroids.

[24] Sinha A., Hollingsworth K.G., Ball S., Cheetham T. (2013). Improving the vitamin D status of vitamin D deficient adults is associated with improved mitochondrial oxidative function in skeletal muscle. J. Clin. Endocrinol. Metab..

[25] de Vries M.A., van der Meulen N., van de Geijn G.M., Klop B., van der Zwan E.M., Prinzen L., Birnie E., Westerman E.M., de Herder W.W., Castro Cabezas M. (2017). Effect of a single dose of vitamin D3 on postprandial arterial stiffness and inflammation in vitamin D-deficient women. J. Clin. Endocrinol. Metab..

[26] Bligh E.G., Dyer W.J. (1959). A rapid method of total lipid extraction and purification. Can. J. Biochem. Physiol..

[27] Chong J., Soufan O., Li C., Caraus I., Li S., Bourque G., Wishart D.S., Xia J. (2018). MetaboAnalyst 4.0: Towards more transparent and integrative metabolomics analysis. Nucleic Acids Res..

[28] Riera-Borrull M., Rodríguez-Gallego E., Hernández-Aguilera A., Luciano F., Ras R., Cuyàs E., Camps J., Segura-Carretero A., Menendez J.A., Joven J. (2016). Exploring the process of energy generation in pathophysiology by targeted metabolomics: Performance of a simple and quantitative method. J. Am. Soc. Mass Spectrom..

[29] Narvaez C.J., Simmons K.M., Brunton J., Salinero A., Chittur S.V., Welsh J.E. (2013). Induction of STEAP4 correlates with 1,25-dihydroxyvitamin D3 stimulation of adipogenesis in mesenchymal progenitor cells derived from human adipose tissue. J. Cell Physiol..

[30] Consiglio M., Viano M., Casarin S., Castagnoli C., Pescarmona G., Silvagno F. (2015). Mitochondrial and lipogenic effects of vitamin D on differentiating and proliferating human keratinocytes. Exp. Dermatol..

[31] Consiglio M., Destefanis M., Morena D., Foglizzo V., Forneris M., Pescarmona G., Silvagno F. (2014). The vitamin D receptor inhibits the respiratory chain, contributing to the metabolic switch that is essential for cancer cell proliferation. PLoS ONE.

[32] Merrill A.H., Sandhoff K., Vance D.E., Vance J.E. (2002). Sphingolipids: metabolism and cell signaling. Biochemistry of Lipids, Lipoproteins.

[33] Okazaki T., Bell R.M., Hannun Y.A. (1989). Sphingomyelin turnover induced by vitamin D3 in HL-60 cells. Role in cell differentiation. J. Biol. Chem..

[34] Wang S., Yang X. (2017). Inter-organ regulation of adipose tissue browning. Cell. Mol. Life Sci..

[35] Zhang X., Ji X., Wang Q., Li J.Z. (2018). New insight into inter-organ crosstalk contributing to the pathogenesis of non-alcoholic fatty liver disease (NAFLD). Protein Cell.

[36] Geilen C.C., Bektas M., Wieder T., Kodelja V., Goerdt S., Orfanos C.E. (1997). 1a,25-Dihydroxyvitamin D3 induces sphingomyelin hydrolysis in HaCaT cells via Tumor Necrosis Factor α. J. Biol. Chem..

[37] Geilen C.C., Bektas M., Wieder T., Orfanos C.E. (1996). The vitamin D, analogue, calcipotriol, induces sphingomyelin hydrolysis in human keratinocytes. FEBS Lett..

[38] Magrassi L., Adorni L., Montorfano G., Rapelli S., Butti G., Berra B., Milanesi G. (1998). Vitamin D metabolites activate the sphingomyelin pathway and induce death of glioblastoma cells. Acta Neurochir..

[39] Nilsson A., Duan R.D. (2006). Absorption and lipoprotein transport of sphingomyelin. J. Lipid Res..

[40] Ohanian J., Ohanian V. (2001). Sphingolipids in mammalian cell signalling. Cell. Mol. Life Sci..

[41] Dressler K.A., Kan C.C., Kolesnick R.N. (1991). Sphingomyelin synthesis is involved in adherence during macrophage differentiation of HL-60 cells. J. Biol. Chem..

[42] Slotte J.P. (2013). Biological functions of sphingomyelins. Prog. Lipid Res..

[43] Fan M., Sidhu R., Fujiwara H., Tortelli B., Zhang J., Davidson C., Walkley S.U., Bagel J.H., Vite C., Yanjanin N.M. (2013). Identification of Niemann-Pick C1 disease biomarkers through sphingolipid profiling. J. Lipid Res..

[44] Schlitt A., Blankenberg S., Yan D., von Gizycki H., Buerke M., Werdan K., Bickel C., Lackner K.J., Meyer J., Rupprecht H.J. (2006). Further evaluation of plasma sphingomyelin levels as a risk factor for coronary artery disease. Nutr. Metab..

[45] Hashemipour S., Sarukhani M.R., Asef zadeh S., Ghazi A.A., Mehrtash B., Ahmadian Yazdi M.H. (2009). Effect of different doses of parenteral vitamin D_3_ on serum 25 (OH) D concentrations. DARU.

[46] Taylor P.N., Davies J.S. (2018). A review of the growing risk of vitamin D toxicity from inappropriate practice. Br. J. Clin. Pharmacol..

[47] Vieth R. (1999). Vitamin D supplementation, 25-hydroxyvitamin D concentrations, and safety. Am. J. Clin. Nutr..

[48] Dalle Carbonare L., Valenti M., del Forno F., Caneva E., Pietrobelli A. (2017). Vitamin D: Daily vs. monthly use in children and elderly—What is going on?. Nutrients.

