# Supplementary material for: Effect of Vitamin D3 on the Postprandial Lipid Profile in Obese Patients: A Non-Targeted Lipidomics Study
Source: Nutrients. 2019 May 27;11(5):1194. doi: 10.3390/nu11051194 (PMC6567161; doi:10.3390/nu11051194)
Supplement: Supplementary file 1 [file nutrients-11-01194-s001.zip › Supplementary Figures.docx]

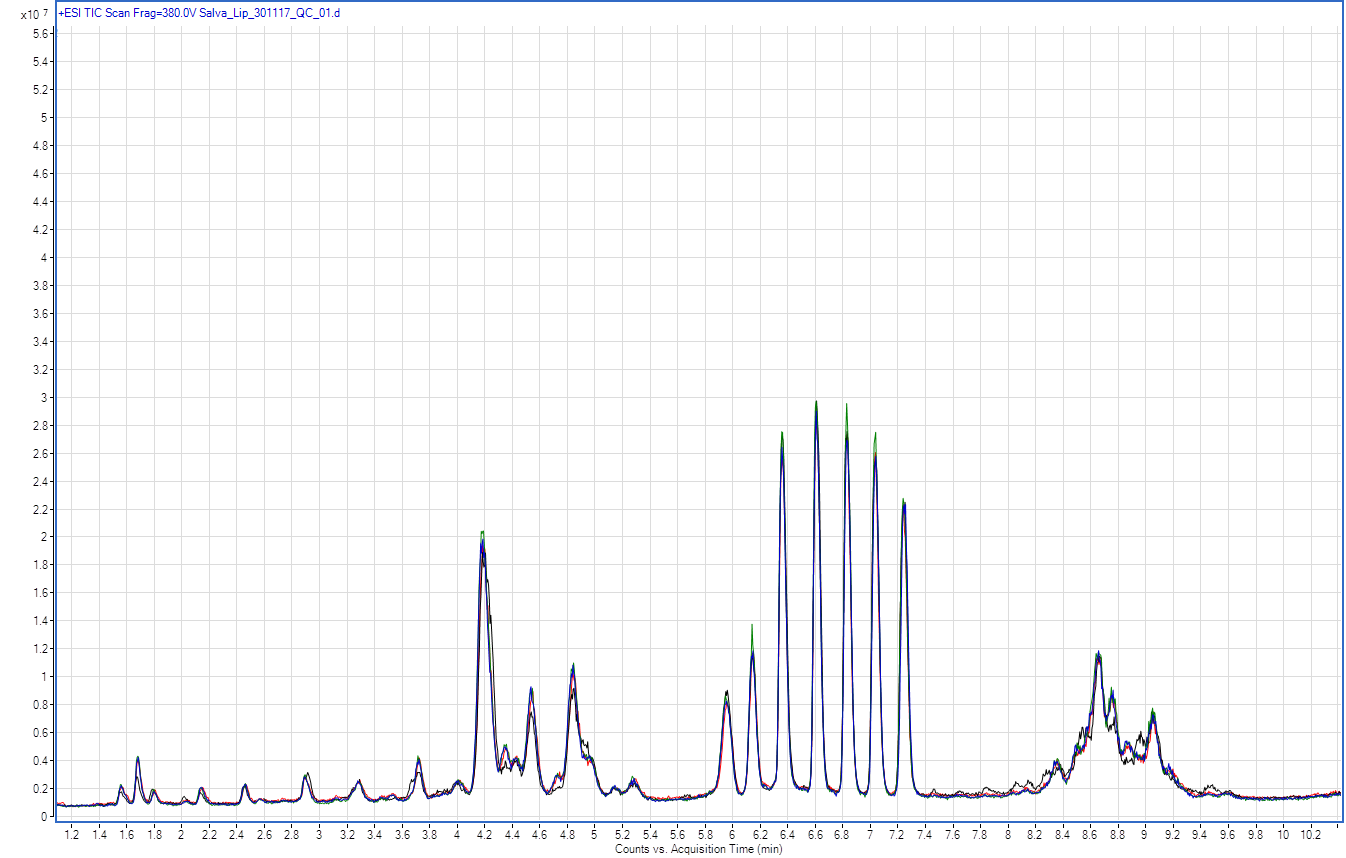


**Supplementary Figure S1.** Overlaid total ion chromatograms of quality controls injected during the analysis of samples.


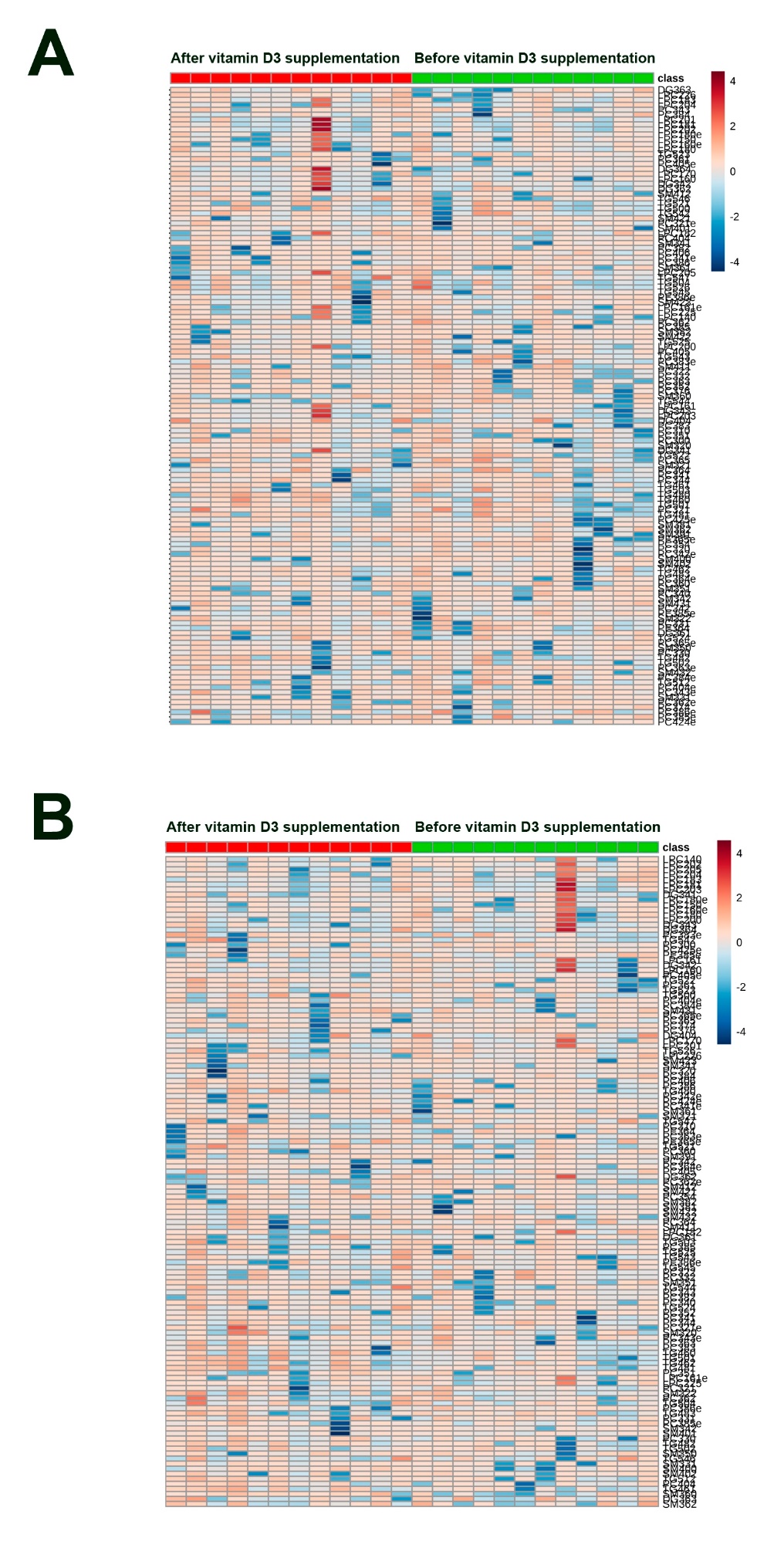


**Supplementary Figure S2.** Heatmap of patients before (green) and after (red) vitamin D_3_ intake in the low (A) and high (B) dose groups.
