# Supplementary figures and images for: Effect of Vitamin D3 on the Postprandial Lipid Profile in Obese Patients: A Non-Targeted Lipidomics Study
Source: Nutrients. 2019 May 27;11(5):1194. doi: 10.3390/nu11051194 (PMC6567161; doi:10.3390/nu11051194)

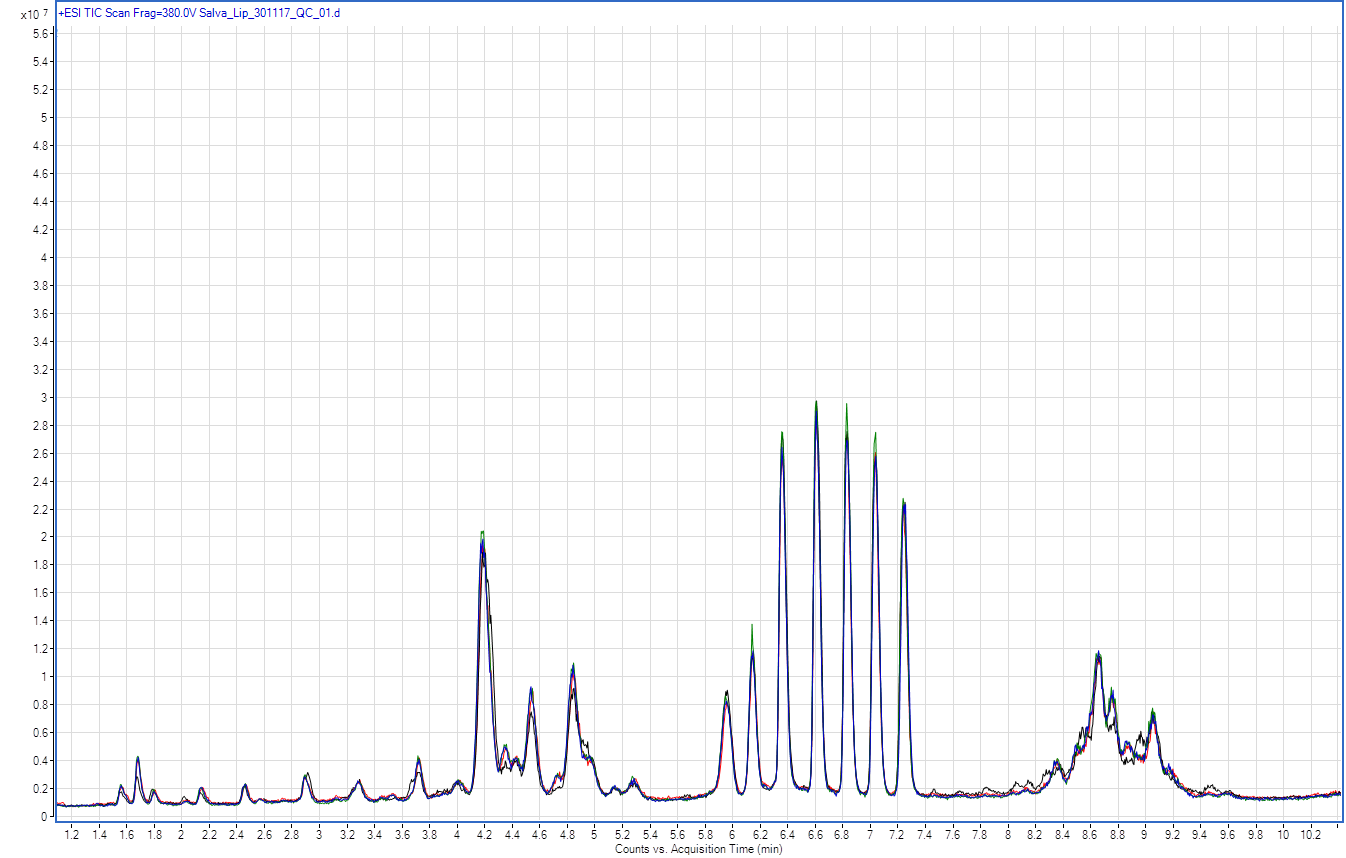

Supplement: Supplementary file 1 [file nutrients-11-01194-s001.zip › Supplementary Figure S1.tif]

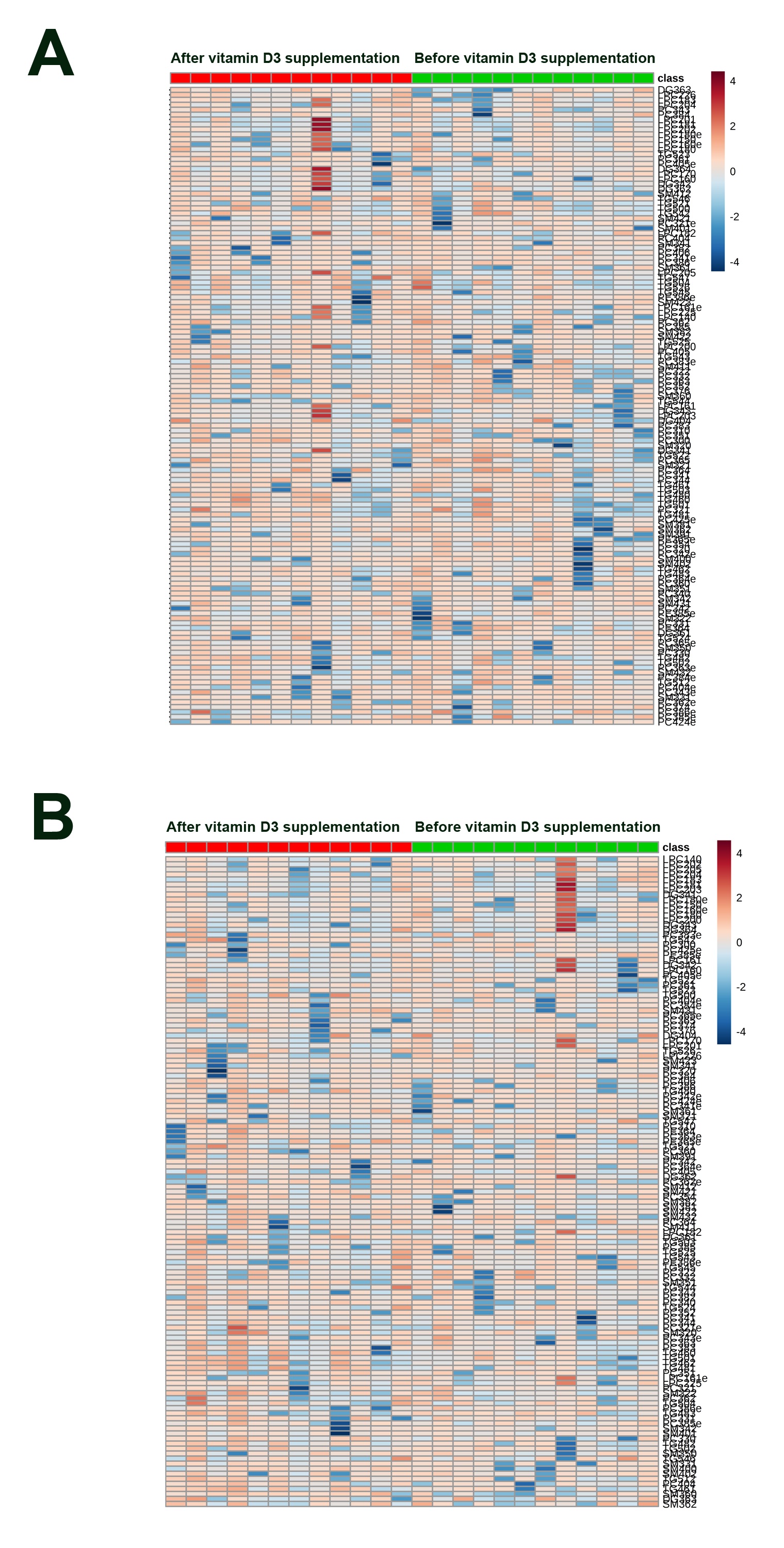

Supplement: Supplementary file 1 [file nutrients-11-01194-s001.zip › Supplementary Figure S2.jpg]
